# Synthesis, Structure Optimization and Antifungal Screening of Novel Tetrazole Ring Bearing Acyl-Hydrazones

**DOI:** 10.3390/ijms130910880

**Published:** 2012-08-30

**Authors:** Maqsood Ahmad Malik, Shaeel Ahmed Al-Thabaiti, Manzoor A. Malik

**Affiliations:** 1 Department of Chemistry, Faculty of Science, King Abdulaziz University, P.O. Box 80203, Jeddah 21413, Saudi Arabia; E-Mail: sthabaiti@kau.edu.sa; 2 Department of Chemistry, Jamia Millia Islamia (Central University), New Delhi, 110025, India; E-Mail: manzoorchem88@gmail.com

**Keywords:** tetrazole, acylhydrazone, *Candida albicans*, flow cytometry, ergosterol

## Abstract

Azoles are generally fungistatic, and resistance to fluconazole is emerging in several fungal pathogens. In an attempt to find novel azole antifungal agents with improved activity, a series of tetrazole ring bearing acylhydrazone derivatives were synthesized and screened for their *in vitro* antifungal activity. The mechanism of their antifungal activity was assessed by studying their effect on the plasma membrane using flow cytometry and determination of the levels of ergosterol, a fungal-specific sterol. Propidium iodide rapidly penetrated a majority of yeast cells when they were treated with the synthesized compounds at concentrations just above MIC, implying that fungicidal activity resulted from extensive lesions of the plasma membrane. Target compounds also caused a considerable reduction in the amount of ergosterol. The results also showed that the presence and position of different substituents on the phenyl ring of the acylhydrazone pendant seem to play a role on the antifungal activity as well as in deciding the fungistatic and fungicidal nature of the compounds.

## 1. Introduction

*Candida albicans* is an opportunistic and often deadly pathogen that invades host tissues, undergoes a dimorphic shift, and then grows as a fungal mass in the kidney, heart or brain. It is the fourth leading cause of hospital-acquired infection in the United States and over 95% of AIDS patients suffer from infections by *C. albicans* [1,2]. *Candida albicans* is the predominant organism associated with candidiasis; but other *Candida* species, including *C. glabrata*, *C. tropicalis* and *C. krusei*, are now emerging as serious nosocomial threats to patient populations [3]. Existing antifungals can treat mucosal fungal infections but very few treatments are available for invasive diseases. The current antifungal therapy suffers from drug related toxicity, severe drug resistance, non-optimal pharmacokinetics, and serious drug-drug interactions. The common antifungal drugs currently used in clinics belong to polyenes and azoles. Polyenes (amphotericin B and nystatin) cause serious host toxicity [4] whereas azoles are fungistatic and their prolonged use contributes to the development of drug resistance in *C. albicans* and other species [5,6]. Because of all these striking problems, there is a pressing need to develop novel antifungal drugs with higher efficiency, broader spectrum, improved pharmacodynamic profiles and lower toxicity.

Various attempts have been undertaken to modify structures of the so far effective azole drugs in order to improve their antimicrobial potency and selectivity [7–9]. However, few reports have discussed the contribution of the tetrazole moiety in such pharmacophore in spite of the potential antibacterial and antifungal activities encountered with some tetrazoles [10–16]. Additionally, it is considered of interest to incorporate other chemotherapeutically-active group within the structure, hoping to impart some synergism to the target compounds. Hydrazones are organic compounds characterized by the presence of –NH–N=CH– group in their molecule. Acyl-hydrazones have an additional donor site like C=O, which determine the versatility and flexibility of these compounds. Such molecules show anticonvulsant, anti-inflammatory, analgesic, antimicrobial, antitumor, anti-platelet, and antiviral properties [17–21]. Some widely used antibacterial drugs such as furacilin, furazolidone and ftivazide are known to contain this group [22]. These observations prompted the incorporation of an acyl-hydrazone moiety into the tetrazole ring to make use of both functionalities in the potentiation of pharmacological activities. Furthermore, after extensive literature search, it was observed that, till date no effort has been made to combine these vital moieties as a single molecular scaffold and to study its antifungal activity. This work constitutes the first attempt to synthesize and assess the antifungal role of tetrazole ring bearing acylhydrazones by studying their effect on morphological aspects of membrane integrity and sterol biosynthesis. Importantly, we also illustrate that these synthesized compounds are effective *in vitro* against *Candida* species which were demonstrated to be largely insensitive to azoles.

## 2. Results and Discussion

### 2.1. Chemistry

**Scheme 1:**
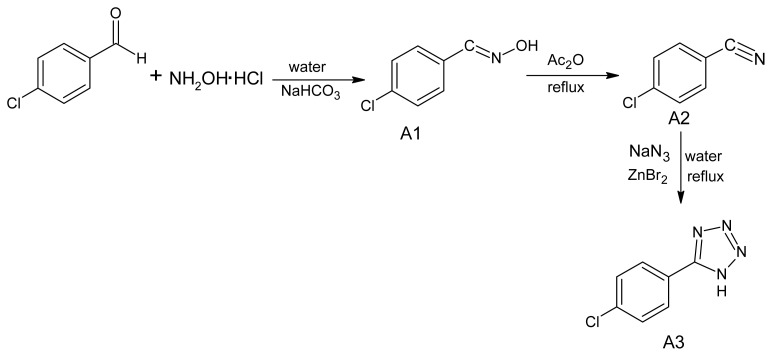
Synthesis of 5-(4-chlorophenyl)-1*H*-tetrazole (**A3**).

**Scheme 2:**
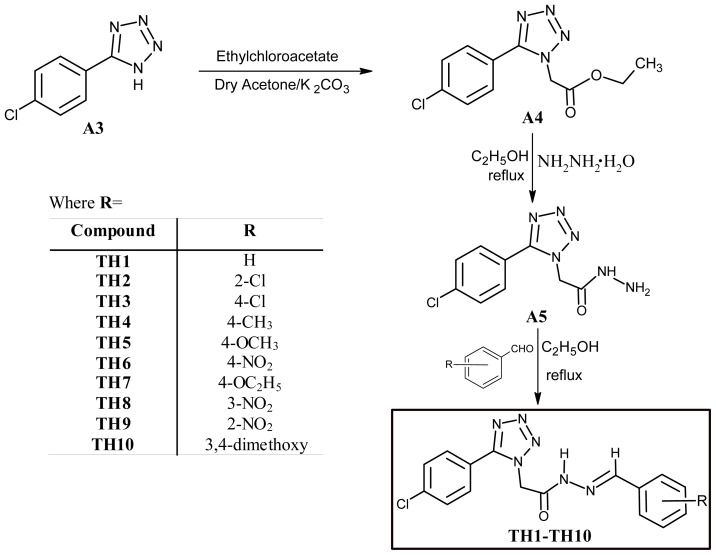
Schematic representation of synthesis of target compounds (**TH1**–**TH10**).

Present study was undertaken to synthesize some novel tetrazole ring bearing acyl-hydrazone derivatives to investigate their probable antifungal effects. Target compounds were obtained in a five step reaction procedure as outlined in Schemes 1 and 2. First of all, 5-(4-chlorophenyl)-1*H*-tetrazole was synthesized from 4-chlorobenzonitrile by following a reported procedure [23]. 4-chlorobenzonitrile in turn was obtained from 4-chlorobenzaldehyde, via an oxime intermediate, following a standard protocol [24]. In the fourth step 2-[5-(4-chlorophenyl)-1*H*-tetrazol-1-yl] acetohydrazide (**A5**) was prepared from 5-(4-chlorophenyl)-1*H*-tetrazole (**A3**) following a reported reaction procedure [25]. The acyl-hydrazone derivatives (**TH1**–**TH10**) were obtained through a condensation reaction of 2-[5-(4-chlorophenyl)-1*H*-tetrazol-1-yl]-acetohydrazide (**A5**) with different aromatic aldehydes in ethanol medium in 1:1 molar ratio. The structure of formed acyl hydrazones was established by elemental analyses, FT-IR, ^1^NMR, ^13^CNMR and ESI-MS spectra.

### 2.2. Biology

#### 2.2.1. MIC and MFC

The Minimum Inhibitory Concentration was defined as the lowest concentration of the compounds (**A1**–**A5** and **TH1**–**TH10**) that causes 80% decrease in absorbance (MIC_80_) compared with that of the control (no test compound). Minimum Fungicidal Concentration (MFC) was defined as the lowest concentration of the test compounds that causes complete death of cells observed by the clear agar plates. Table 1 summarizes the *in vitro* susceptibilities (MIC and MFC) of 3 standard laboratory *Candida* isolates, 11 clinically obtained *Candida* isolates and 3 resistant strains (Table 2) against all the test compounds. MIC range of reference drugs is also reported in the table legend. Most clinical strains of *Candida* spp. were shown to be susceptible to the tested compounds at doses comparable with reference antifungal drugs. It is interesting to note that compounds **TH3**–**TH7** showed fungicidal activity while compounds **TH1**, **TH8**–**TH10** as expected showed fungistatic activity as that of the most commonly used antifungal fluconazole. Compound **TH2** is inactive against all the tested isolates of *Candida*.

#### 2.2.2. Disc Diffusion Halo Assays

Table 1 shows i*n vitro* sensitivity of all the test compounds against the various *Candida* isolates. Index of sensitivity (SI) was defined as the ratio of diameter of Zone of Inhibition (ZOI in mm) to the concentration of each drug (mg mL^−1^). The sequence of the SI for all the test compounds decreases in the order given below:

TH4≥TH9≥TH10>TH7≥TH1>TH3>TH5≥TH8>TH6

While variability may occur among the antifungal activity of the test compounds, it is exciting that some of the compounds (**TH3**, **TH4**, **TH5**, **TH6** and **TH7**) showed fungicidal potential, as is evident from the clear zones of inhibition in disc diffusion assay; whereas in contrast other test compounds (**TH1**, **TH8**, **TH9** and **TH10**) showed a visible turbid halo indicating their fungistatic nature. There was a complete growth observed around the disc impregnated with compound **TH2**, which indicates that compound **TH2** do not have any anticandidal activity. Additional *in vivo* tests are required to be performed on new fungal strains and other pathogens to evaluate antimicrobial potential of these novel compounds.

#### 2.2.3. Confocal Microscopy Analysis

**Figure 1:**
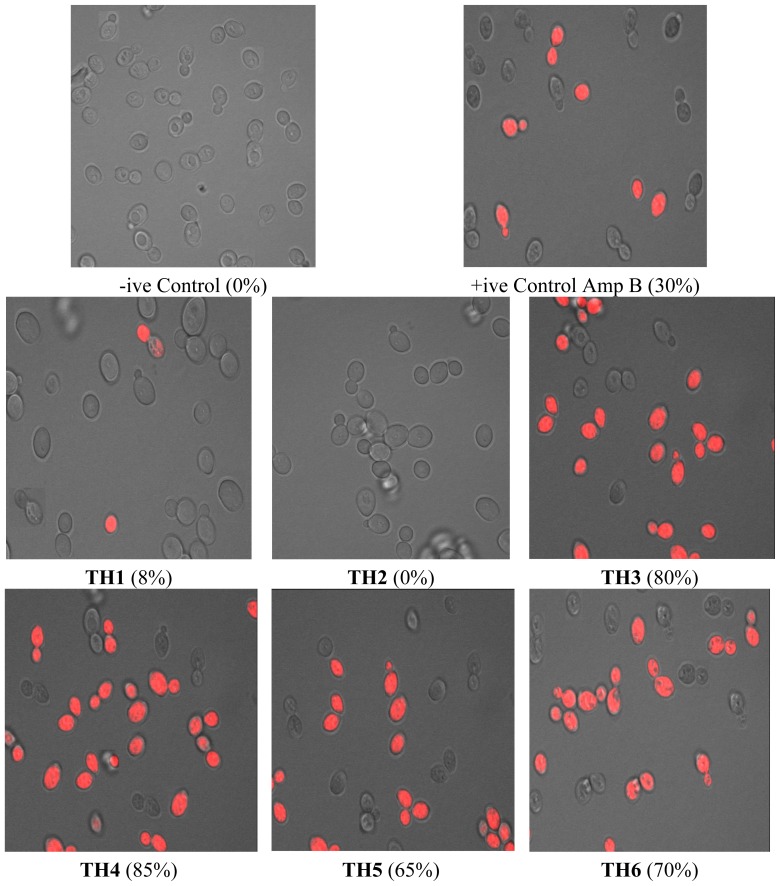

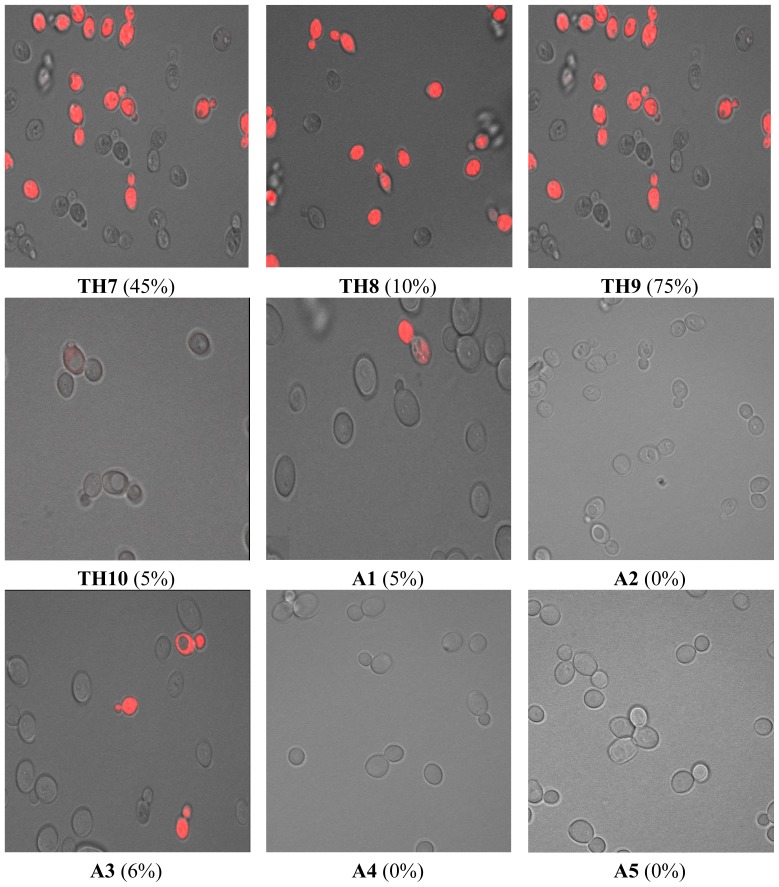
Laser confocal images of *Candida* cells. Cells with membrane damage are seen stained with PI (red signals).

*Candida* cells were grown and then stained with propidium iodide (PI) as described in materials and methods. PI penetrates only those cells which have severe membrane lesions; the entire yeast cell appears red. The laser confocal images of stained *Candida* cells exposed to test compounds are shown in Figure 1.

Our results showed that PI penetrates over 80% of the yeast cells when treated with 4× MIC of compound **TH3** and **TH4**, indicating that the structure of the cell membrane was disrupted by these test compounds to a large extent. Similarly, compounds **TH5**, **TH6**, **TH7** and **TH9** also showed disruption of membrane integrity ranging from 45% to 75% of cells. A similar exposure to compounds **TH1**, **TH8** and **TH10** (4× MIC) induced permeation of PI in less than 10% of the cells (Figure 1), indicating that these compounds have mechanism of antimicrobial activity different than membrane disruption. Under these conditions, 2 mg L^−1^ AmB (MIC range: 0.125–2.0 mg L^−1^) induced PI staining in less than 30% of *Candida* cells (Figure 1). Compound **TH2** and intermediates **A2**, **A3**, **A4** and **A5** did not show any membrane disruption at these concentrations, which directly corresponds to the growth results. The number of PI +ve cells and of non-viable yeast cells correlated well.

#### 2.2.4. Sterol Quantitation

**Figure 2:**
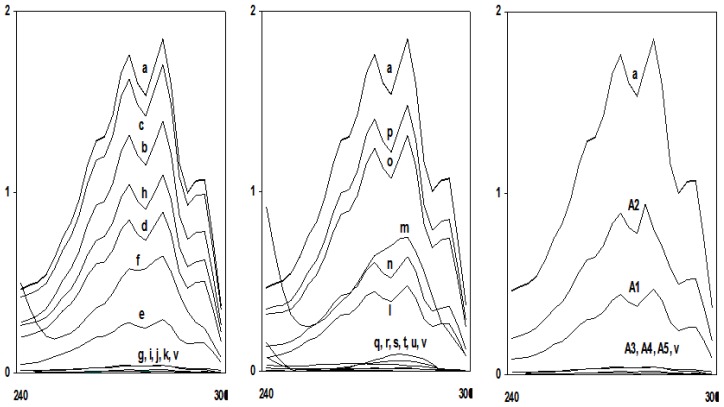
UV spectrophotometric sterol profiles of representative susceptible *Candida* isolates. Isolates were grown for 16 h in sabouraud dextrose broth containing 0 (curve a), ½ MIC’s of compounds—**TH1** (curve b), **TH2** (curve c), **TH3** (curve d), **TH4** (curve e), **TH5** (curve f), **TH6** (curve l), **TH7** (curve m), **TH8** (curve n), **TH9** (curve o), **TH10** (curve p) or MIC’s of compounds **TH1** (curve g), **TH2** (curve h), **TH3** (curve i), **TH4** (curve j), **TH5** (curve k), **TH6** (curve q), **TH7** (curve r), **TH8** (curve s), **TH9** (curve t), **TH10** (curve u) or 64 mg of fluconazole per L (curve v). Curves **A1**, **A2**, **A3**, **A4** and **A5** represent cells treated with 125 mg L^−1^ of their respective intermediates. Sterols were extracted from cells, and spectral profiles between 240 and 300 nm were determined. *X*-axis represents the wavelength between 240 nm and 300 nm.

**Figure 3:**
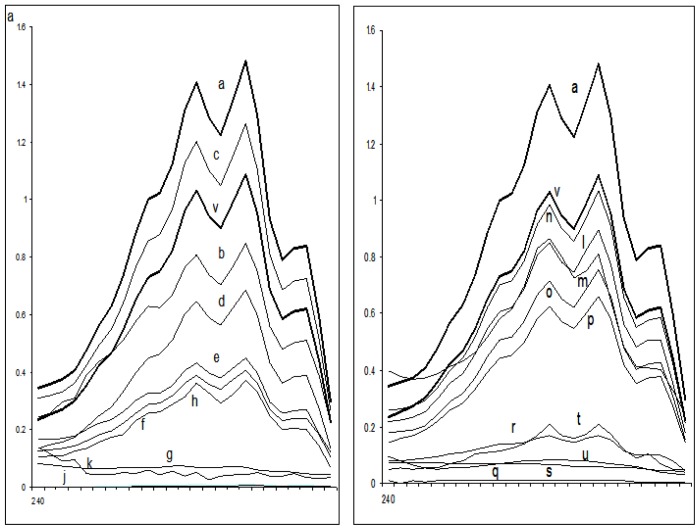
UV spectrophotometric sterol profiles of representative resistant *Candida* isolates. Isolates were grown for 16 h in sabouraud dextrose broth containing 0 (curve a), ½ MIC’s of compounds—**TH1** (curve b), **TH2** (curve c), **TH3** (curve d), **TH4** (curve e), **TH5** (curve f), **TH6** (curve l), **TH7** (curve m), **TH8** (curve n), **TH9** (curve o), **TH10** (curve p) or MIC’s of compounds—**TH1** (curve g), **TH2** (curve h), **TH3** (curve i), **TH4** (curve j), **TH5** (curve k), **TH6** (curve q), **TH7** (curve r), **TH8** (curve s), **TH9** (curve t), **TH10** (curve u) or 64 mg of fluconazole per liter (curve v). Sterols were extracted from cells, and spectral profiles between 240 and 300 nm were determined. *X*-axis represents the wavelength between 240 nm and 300 nm.

Table 3 summarizes the effect of the test compounds on ergosterol biosynthesis in different fluconazole-sensitive and fluconazole-resistant *Candida* isolates. The total ergosterol content was determined for each isolate grown in ½ MIC and MIC values of all the test compounds. A dose-dependent decrease in ergosterol production was observed when isolates were grown in the presence of test compounds (Figures 2 and 3).

Ergosterol content decreased by >95% when *Candida* cells were exposed to MIC values of all the test compounds except compound **TH2** where only 41% of ergosterol biosynthesis inhibition was observed at the concentration of 64 mg L^−1^. In case of resistant isolates, ergosterol content decreased to 85%–95% when cells were treated with MIC concentrations of the test compounds. The decrease in the total cellular ergosterol content for susceptible isolates ranged from 25% to 75% after exposure to ½ MIC values of the test compounds. Similarly in case of resistant isolates, 40%–76% ergosterol decrease was observed at ½ MIC values. It was also observed that a very significant inhibition of ergosterol biosynthesis was observed even at the sub-MIC concentrations of these tetrazoles. Interestingly, the ergosterol biosynthesis inhibition by the intermediates **A1** and **A2** was only 75% and 49% at the concentration which is 4 times more than the MIC values of these intermediates. At the same concentration, **A3**, **A4** and **A5** decreased more than 95% ergosterol biosynthesis. The decrease in total cellular ergosterol content of sensitive cells treated with fluconazole was measured to be 100%.

Despite the introduction of improved antifungal drugs for treatment and prophylaxis, invasive fungal infections remain a significant clinical problem. Infections caused by eukaryotic organisms like yeasts generally present more difficult therapeutic problems than do bacterial infections. There are relatively few antifungal agents that can identify unique targets not shared with human hosts. The fungal cell wall may be considered to be a prime target for selectively toxic antifungal agents because of its chitin structure, absent from human cells. Azoles have long been an investigatory class of compounds owing to their therapeutic importance. The present work is an attempt to understand the mechanism of antifungal activity of newly synthesized tetrazole ring bearing acylhydrazone derivatives and the intermediate compounds against *Candida* species as a prerequisite for its application in the treatment of mucocutaneous infections or combinatorial therapies. Most therapies, designed to treat fungal infections, target the ergosterol biosynthesis pathway or its end product, ergosterol. Ergosterol is the main sterol of yeasts and other fungi, and thus is necessary for growth and normal membrane function of cells. Besides serving as a bioregulator of membrane fluidity, asymmetry and membrane integrity, ergosterol contributes to the proper function of membrane-bound enzymes [26]. Azoles, including fluconazole, the most commonly used drug for treatment and prevention of candidiasis, target lanosterol 14α-demethylase, one of the enzymes involved in biosynthesis of ergosterol. In this study an attempt was made to study the effect of the target compounds on the ergosterol biosynthesis. Analysis of sterols obtained from tested *Candida* strains showed no major differences in either the sterol content or the sterol pattern when untreated. In contrast, growth in the presence of sub-inhibitory and inhibitory concentrations of target compounds altered the sterol patterns of the all the tested strains. Studies on the effects of these compounds at various sub-inhibitory concentrations on the sterols of the *Candida* strains showed that they act in a dose-dependent manner to decrease ergosterol content (Figures 2 and 3). Effects on membrane integrity thus appear to originate from inhibition of ergosterol biosynthesis in a manner similar to that of fluconazole. The antifungal effects were attributed to the ability of the target compounds to inhibit ergosterol biosynthesis and to make the cytoplasmic membrane porous in both drug-sensitive as well as drug resistant isolates. The latter, in turn, could also be due to decreased sterol biosynthesis. Yet other mechanisms, including extensive lipid peroxidation due to oxidative stress, cannot be ruled out as a reason for the incorporation of PI leading to cell death.

A close examination of the structures of the active compounds presented in Table 2 revealed that their antimicrobial activity is strongly bound to the nature and position of the substituent on the phenyl ring of the acylhydrazone pendent. Parent compound (**A1**) and the intermediate compounds (**A2**–**A5**) however did not show any appreciable activity. Preliminary SAR campaign showed that the replacement of hydrogen in **TH1** by a chlorine group in **TH3** resulted in a 10 fold decrease in activity. Introduction of the same chlorine group at C2 of the phenyl ring in **TH2** resulted in complete absence of activity. Introduction of different electron withdrawing and electron donating groups at the C4 and C2 position of the phenyl ring also showed an interesting pattern of activity, where activity seems to be guided not only by the electron withdrawing and donating nature of the substituents but also by the ring activating and deactivating nature of the substituents. Presence of a nitro group at C3 in compound TH8 showed positive effect on the activity and similar results were obtained when the group was introduced at C2 in **TH9**. Presence of two methoxy groups at C3 and C4 position in compound **TH10** showed less activity than compounds **TH8** and **TH9**. From the results it can be clearly documented that the position of substituents determines the fungistatic and fungicidal nature of the compounds, where presence of substituents at position C4 makes the compound fungicidal, whereas either the absence of substituents at C4 or the presence of substituents at C3 makes the compound fungistatic. Interestingly the presence of a substituent at C3 and C4 in a same compound as in **TH10** also makes the compound fungistatic. In general, it could be clearly recognized that potential antifungal activity as well as the fungistatic and fungicidal nature of the compounds is structure dependant. However the structure of the synthesized compounds needs to be further optimized so that more insight into the structure-activity relationship of the compounds gets revealed.

## 3. Materials and Methods

Solvents and organic reagents were purchased from Sigma Aldrich, Merck (Germany) and Loba Chemie (India) and were used without further purification. Melting points (mp) were measured using a Mel-temp instrument, and the results are uncorrected. Elemental analyses were performed on HeraeusVario EL III analyzer. The results were within ±0.4% of the theoretical values. IR spectra were recorded on Perkin-Elmer model 1600 FT-IR RX1 spectrophotometer as KBr discs/ATR mode. ^1^H NMR and ^13^C NMR spectra were recorded on Bruker AVANCE 300 (300.13) MHz spectrometer using DMSO-d_6_/CDCl_3_ as solvent with TMS as internal standard. Splitting patterns are designated as follows; s, singlet; d, doublet; dd, doublet of doublets; t, triplet; m, multiplet. Chemical shift values are given in ppm. ESI-MS was recorded on a MICROMASS QUATTRO II triple quadrupole mass spectrometer. Reactions were monitored using thin-layer chromatography (TLC) using commercially available precoated plates (Merck Kieselgel 60 F_254_ silica). Visualization was achieved with UV light at 254 nm or I_2_ vapor staining.

### 3.1. Synthesis of 5-(4-Chlorophenyl)-1*H*-tetrazole (A3)

5-(4-chlorophenyl)-1*H*-tetrazole was synthesized from 4-chlorobenzonitrile by following a reported procedure [23]. 4-chlorobenzonitrile in turn was obtained from 4-chlorobenzaldehyde, following a reported procedure [24]. Nitrile (2.66 g, 20 mmol), sodium azide (1.43 g, 22 mmol) and zinc bromide (4.50 g, 20 mmol), were put in 60 mL of water. Five microliter of isopropanol was also added to stop the formation of clumps. The reaction mixture was refluxed for 24 h and monitored by TLC; vigorous stirring is essential. After 24 h HCl (3 N, 30 mL) and ethyl acetate (100 mL) were added, and vigorous stirring was continued until no solid was present and the aqueous layer had a pH of 1. If necessary, additional ethyl acetate was added. The organic layer was isolated and the aqueous layer extracted with ethyl acetate. The combined organic layers were evaporated, 250 mL of 0.25 N NaOH was added, and the mixture was stirred for 15 min, until the original precipitate was dissolved and a suspension of zinc hydroxide was formed. The suspension was filtered, and the solid washed with 50 mL of 1 N NaOH. To the filtrate was added 50 mL of 3 N HCl with vigorous stirring causing the tetrazole to precipitate. The tetrazole was filtered and washed with 100 mL of 3 N HCl and dried in a drying oven to furnish the tetrazole as a white powder.

Yield 80%; mp 172–175 °C; IR *ν*_max_ (cm^−1^): 3289 (NH), 3018 (C–H, Ar), 1622 (C=N), 1574 (C=C, Ar), 798 (C–Cl); ^1^H NMR (DMSO) δ (ppm): 8.74 (1H, NH, br s), 7.65–7.40 (4H, m); ^13^C NMR (DMSO) δ (ppm): 156.3 (C=N), 138.5, 129.4, 126.3; ESI-MS *m*/*z*: [M+H]^+^ 182.03.

### 3.2. Synthesis of ethyl [5-(4-Chlorophenyl)-tetrazol-1-yl]acetate (A4)

A mixture of 5-(4-chlorophenyl)-1*H*-tetrazole (**A3**) (1 mmol), ethyl chloroacetate (1 mmol) and potassium carbonate (1.5 mmol) in dry acetone (5–10 mL) was refluxed for 50 h. The reaction mixture was filtered hot and the solvent was distilled off from the filtrate. The crude ester thus obtained was purified by recrystallization from ethanol.

Yield 62.5%; mp 158–160 °C; IR *ν*_max_ (cm^−1^): 3028 (C–H, Ar), 1722 (C=O), 1635 (C=N), 1584 (C=C, Ar), 758 (C–Cl); ^1^H NMR (DMSO) δ (ppm): 7.98–7.45 (4H, m), 4.82 (2H, CH_2_, s), 4.11 (2H, CH_2_, m); 1.38 (3H, CH_3_, t); ^13^C NMR (DMSO) δ (ppm): 162.2 (C=O) 154.5 (C=N), 137.7, 126.0, 124.8, 122.0, 118.2, 64.5, 48.2, 16.2; ESI-MS *m*/*z*: [M+H]^+^ 267.06.

### 3.3. Synthesis of 2-[5-(4-Chlorophenyl)-tetrazol-1-yl]acetohydrazide (A5)

2-[5-(4-chlorophenyl)-tetrazol-1-yl]acetohydrazide was prepared by a reported method [25]. Yield 65%; mp 188–190 °C; IR *ν*_max_ (cm^−1^): 3280 (NH), 3022 (C–H, Ar), 1711 (C=O), 1628 (C=N), 1595 (C=C, Ar), 784 (C–Cl); ^1^H NMR (DMSO) δ (ppm): 11.26 (1H, NH, s), 7.45–7.22 (4H, m), 4.46 (2H, CH_2,_ s), 2.64 (2H, s, NH_2_); ^13^C NMR (DMSO) δ (ppm): 164.7 (C=O), 154.0 (C=N), 130.4, 129.5, 128.0, 68.5; ESI-MS *m*/*z*: [M+H]^+^ 254.05.

### 3.4. Experimental Procedure for the Synthesis of Acyl-hydrazones (TH1–TH10)

To a stirred solution of hydrazide **A5** (1 mmol) and different aromatic aldehydes (1 mmol) in ethanol (25 mL) was added water (5 mL) followed by the dropwise addition of glacial acetic acid (0.2 mL). The resulting mixture was refluxed for 5 h, after which the solution was poured into ice water. The mixture was stirred until a precipitate formed, which was collected using suction filtration and dried, followed by recrystallization in aqueous methanol, giving acyl-hydrazones (**TH1**–**TH10**) in varying yields (74%–85%).

### 3.5. 2-[5-(4-Chlorophenyl)-tetrazol-1-yl]-*N*′-[(E)-phenylmethylidene]-acetohydrazide (TH1)

White solid; Yield 82%; mp 238–240 °C; Anal. Calc. For C_16_H_13_N_6_OCl: C 56.39%, H 3.85%, N 24.66% found: C 56.49%, H 4.03%, N 24.59%; IR *ν*_max_ (cm^−1^): 3375 (NH), 3078, 2855 (C–H), 1667 (C=O), 1621–1465 (C=N and C=C), 785 (C–Cl); ^1^H NMR (DMSO-*d*_6_) δ (ppm): 11.76 (1H, br, NH), 8.45 (1H, s, –N=CH–), 7.87–7.24 (9H, m, Ar–H), 5.52 (2H, s, methylenic); ^13^C NMR (DMSO-*d*_6_) δ (ppm): 170.9 (C=O), 154.0 (C=N), 145.5, 134.4, 132.0, 130.5, 129.8 129.1, 128.5, 128.2, 49.5; ESI-MS *m*/*z*: [M+H]^+^ 341.09.

### 3.6. *N*′-[(E)-(2-Chlorophenyl)methylidene]-2-[5-(2-chlorophenyl)-tetrazol-1-yl]-acetohydrazide (TH2)

White solid; Yield 78%; mp 218–220 °C; Anal. Calc. For C_16_H_12_N_6_OCl_2_: C 51.22%, H 3.22%, N 22.40% found: C 51.34%, H 3.08%, N 22.52%; IR *ν*_max_ (cm^−1^): 3354 (NH), 3045, 2860 (C–H), 1701 (C=O), 1644–1467 (C=N and C=C), 764 (C–Cl); ^1^H NMR (DMSO-*d*_6_) δ (ppm): 11.45 (1H, br, NH), 8.22 (1H, s, –N=CH–), 7.75–7.14 (8H, m, Ar–H), 5.49 (2H, s, methylenic); ^13^C NMR (DMSO-*d*_6_) δ (ppm): 170.2 (C=O), 158.6 (C=N), 143.5, 134.5, 133.2, 132.1, 130.5, 129.4 128.9, 128.4, 49.8; ESI-MS *m*/*z*: [M+H]^+^ 375.05.

### 3.7. *N*′-[(E)-(2-Chlorophenyl)methylidene]-2-[5-(4-chlorophenyl)-tetrazol-1-yl]-acetohydrazide (TH3)

White solid; Yield 80%; mp 225–228 °C; Anal. Calc. For C_16_H_12_N_6_OCl_2_: C 51.22%, H 3.22%, N 22.40% found: C 51.28%, H 3.14%, N 22.48%; IR *ν*_max_ (cm^−1^): 3358 (NH), 3058, 2862 (C–H), 1710 (C=O), 1654–1482 (C=N and C=C), 780 (C–Cl); ^1^H NMR (DMSO-*d*_6_) δ (ppm): 11.49 (1H, br, NH), 8.20 (1H, s, –N=CH–), 7.96–7.00 (8H, m, Ar–H), 5.44 (2H, s, methylenic); ^13^C NMR (DMSO-*d*_6_) δ (ppm): 170.2 (C=O), 160.5 (C=N), 143.3, 137.2, 134.8, 132.0, 131.2, 129.6 128.5, 128.0, 48.5; ESI-MS *m*/*z*: [M+H]^+^ 375.05.

### 3.8. 2-[5-(4-Chlorophenyl)-tetrazol-1-yl]-*N*′-[(E)-(4-ethylphenyl)methylidene]-acetohydrazide (TH4)

White solid; Yield 74%; mp 230–232 °C; Anal. Calc. For C_17_H_15_N_6_OCl: C 57.55%, H 4.26%, N 23.69%, found: C 57.42%, H 4.34%, N 23.61%; IR *ν*_max_ (cm^−1^): 3348 (NH), 3030, 2859 (C–H), 1690 (C=O), 1645–1468 (C=N and C=C), 775 (C–Cl); ^1^H NMR (DMSO-*d*_6_) δ (ppm): 11.94 (1H, br, NH), 8.23 (1H, s, –N=CH–), 7.76–7.28 (8H, m, Ar–H), 5.61 (2H, s, methylenic), 2.82 (3H, s, CH_3_); ^13^C NMR (DMSO-*d*_6_) δ (ppm): 172.8 (C=O), 158.5 (C=N), 143.7, 140.2, 134.8, 130.8, 129.6, 129.0, 128.5, 128.0, 48.7, 24.6; ESI-MS *m*/*z*: [M+H]^+^ 355.10.

### 3.9. 2-[5-(4-Chlorophenyl)-tetrazol-1-yl]-*N*′-[(E)-(4-methoxyphenyl)methylidene]-acetohydrazide (TH5)

White solid; Yield 78%; mp 236–238 °C; Anal. Calc. For C_17_H_15_N_6_O_2_Cl: C 55.07%, H 4.08%, N 22.66%, found: C 55.16%, H 4.22%, N 22.67%; IR *ν*_max_ (cm^−1^): 3355 (NH), 3035, 2853 (C–H), 1686 (C=O), 1658–1450 (C=N and C=C), 768 (C–Cl); ^1^H NMR (DMSO-*d*_6_) δ (ppm): 11.77 (1H, br, NH), 8.21 (1H, s, –N=CH–), 7.86–7.02 (8H, m, Ar–H), 5.24 (2H, s, methylenic), 3.80 (3H, s, OCH_3_); ^13^C NMR (DMSO-*d*_6_) δ (ppm): 173.1 (C=O), 157.4 (C=N), 155.8, 143.0, 140.2, 134.2, 130.2, 129.4, 128.5, 122.6, 56.8, 48.7; ESI-MS *m*/*z*: [M+H]^+^ 371.10.

### 3.10. 2-[5-(4-Chlorophenyl)-tetrazol-1-yl]-*N*′-[(E)-(4-nitrophenyl)methylidene]-acetohydrazide (TH6)

White solid; Yield 85%; mp 238–240 °C; Anal. Calc. For C_16_H_12_N_7_O_3_Cl: C 49.82%, H 3.14%, N 25.42%, found: C 49.82%, H 3.24%, N 25.50%; IR *ν*_max_ (cm^−1^): 3370 (NH), 3065, 2850 (C–H), 1680 (C=O), 1655–1458 (C=N and C=C), 1557, 1360 (–NO_2_), 785 (C–Cl); ^1^H NMR (DMSO-*d*_6_) δ (ppm): 11.54 (1H, br, NH), 8.38 (1H, s, –N=CH–), 7.97–7.22 (8H, m, Ar–H), 5.19 (2H, s, methylenic); ^13^C NMR (DMSO-*d*_6_) δ (ppm): 173.1 (C=O), 157.4 (C=N), 155.8, 143.0, 140.2, 134.2, 130.2, 129.4, 128.5, 122.6, 56.8, 48.7; ESI-MS *m*/*z*: [M+H]^+^ 386.07.

### 3.11. 2-[5-(4-Chlorophenyl)-tetrazol-1-yl]-*N*′-[(E)-(4-ethoxyphenyl)methylidene]-acetohydrazide (TH7)

White solid; Yield 76%; mp 240–242 °C; Anal. Calc. For C_18_H_17_N_6_O_2_Cl: C 56.18%, H 4.45%, N 21.84%, found: C 56.26%, H 4.48%, N 22.04%; IR *ν*_max_ (cm^−1^): 3363 (NH), 3049, 2855 (C–H), 1693 (C=O), 1665–1450 (C=N and C=C), 773 (C–Cl); ^1^H NMR (DMSO-*d*_6_) δ (ppm): 11.73 (1H, br, NH), 8.29 (1H, s, –N=CH–), 7.85–7.10 (8H, m, Ar–H), 5.22 (2H, s, methylenic), 3.95–2.87 (2H, m, OCH_2_CH_3_), 1.35 (3H, t, OCH_2_CH_3_); ^13^C NMR (DMSO-*d*_6_) δ (ppm): 170.3 (C=O), 159.6 (C=N), 156.3, 148.9, 143.5, 136.8, 134.3, 132.4, 130.2, 129.6, 128.4, 118.2, 68.7, 47.8, 14.9; ESI-MS *m*/*z*: [M+H]^+^ 385.11.

### 3.12. 2-[5-(4-Chlorophenyl)-tetrazol-1-yl]-*N*′-[(E)-(3-nitrophenyl)methylidene]-acetohydrazide (TH8)

White solid; Yield 80%; mp 215–218 °C; Anal. Calc. For C_16_H_12_N_7_O_3_Cl: C 49.82%, H 3.14%, N 25.42%, found: C 49.88%, H 3.26%, N 25.49%; IR *ν*_max_ (cm^−1^): 3355 (NH), 3035, 2856 (C–H), 1688 (C=O), 1660–1455 (C=N and C=C), 1545, 1365 (–NO_2_), 778 (C–Cl); ^1^H NMR (DMSO-*d*_6_) δ (ppm): 11.83 (1H, br, NH), 8.17 (1H, s, –N=CH–), 8.02–7.37 (8H, m, Ar–H), 5.18 (2H, s, methylenic); ^13^C NMR (DMSO-*d*_6_) δ (ppm): 170.3 (C=O), 159.6 (C=N), 148.9, 143.5, 136.8, 134.3, 132.4, 130.2, 129.6, 128.4, 121.2, 47.8; ESI-MS *m*/*z*: [M+H]^+^ 386.07.

### 3.13. 2-[5-(4-Chlorophenyl)-tetrazol-1-yl]-*N*′-[(E)-(2-nitrophenyl)methylidene]-acetohydrazide (TH9)

White solid; Yield 75%; mp 210–212 °C; Anal. Calc. For C_16_H_12_N_7_O_3_Cl: C 49.82%, H 3.14%, N 25.42%, found: C 49.92%, H 3.28%, N 25.48%; IR *ν*_max_ (cm^−1^): 3362 (NH), 3045, 2855 (C–H), 1687 (C=O), 1662–1459 (C=N and C=C), 1552, 1360 (–NO_2_), 784 (C–Cl); ^1^H NMR (DMSO-*d*_6_) δ (ppm): 11.76 (1H, br, NH), 8.20 (1H, s, –N=CH–), 8.04–7.22 (8H, m, Ar–H), 5.20 (2H, s, methylenic); ^13^C NMR (DMSO-*d*_6_) δ (ppm): 172.8 (C=O), 158.2 (C=N), 148.6, 143.0, 135.4, 134.3, 132.0, 131.5, 130.5, 128.6, 125.4, 120.0, 48.2; ESI-MS *m*/*z*: [M+H]^+^ 386.07.

### 3.14. 2-[5-(4-Chlorophenyl)-tetrazol-1-yl]-*N*′-[(E)-(3,4-dimethoxyphenyl)methylidene]-acetohydrazide (TH10)

White solid; Yield 85%; mp 222–225 °C; Anal. Calc. For C_18_H_17_N_6_O_3_Cl: C 53.94%, H 4.27%, N 20.97%, found: C 54.08%, H 4.38%, N 21.14%; IR *ν*_max_ (cm^−1^): 3360 (NH), 3033, 2865 (C–H), 1705 (C=O), 1654–1459 (C=N and C=C), 795 (C–Cl); ^1^H NMR (DMSO-*d*_6_) δ (ppm): 11.62 (1H, br, NH), 8.24 (1H, s, –N=CH–), 7.74–7.15 (7H, m, Ar–H), 5.24 (2H, s, methylenic), 3.70 (6H, s, OCH_3_); ^13^C NMR (DMSO-*d*_6_) δ (ppm): 172.8 (C=O), 158.2 (C=N), 152.0, 149.2, 143.5, 134.6, 129.8, 128.6, 128.0, 122.5, 116.2, 114.8, 58.0, 48.2; ESI-MS *m*/*z*: [M+H]^+^ 401.11.

## 4. Antifungal Studies

### 4.1. Strains and Media

Fungal strains used in the present study have been listed in Table 2. The clinical isolates were collected from All India Institute of Medical Sciences and VMMC Safdarjung Hospital, New Delhi, India. All strains were grown on Yeast extract (1% *w*/*v*), Peptone (2%), Dextrose (2%); (YPD) medium. Cultures were maintained on YPD agar plates. Fluconazole (FLC) and Amphotericin B (AmB) were procured from HiMedia. All inorganic chemicals were of analytical grade and procured from E. Merck (India).

### 4.2. Determination of MIC and MFC

The Minimum Inhibitory Concentration (MIC) was defined as the lowest concentration of the test compounds that causes inhibition of visible growth of *Candida* cells. MIC_80_ was determined *in vitro* in liquid medium by macro broth dilution method as described by CLSI reference document M27-A3 (CLSI, 2008) for 14 *Candida* isolates (Table 2). To determine minimum fungicidal concentration (MFC) values, after reading the corresponding MIC values, 20 μL samples from all optically clear tubes (complete growth inhibition) plus the last tube showing growth were subcultured on YEPD agar plates. The plates were incubated at 35 °C for a minimum of 3 days, until growth was clearly visible in the control samples, and MFC values were determined as the lowest concentration of the test compounds for which there was no visible growth.

### 4.3. Disc Diffusion Halo Assays

Strains were inoculated into liquid YPD medium and grown overnight at 37 °C. The cells were then pelleted and washed three times with distilled water. Approximately 10^5^ cells/mL were inoculated in molten agar media at 40 °C and poured into 100-mm-diameter Petri plates. Filter discs were kept on solid agar and test compounds were spotted on the disc. Test compounds (4 fold more than MIC) dissolved in DMSO, or solvent control (DMSO) were pipetted onto 4-mm-diameter filter disc. Fluconazole (10 mg/L) was also applied on the discs to serve as negative control. The diameter of zone of inhibition was recorded in millimeters after 48 h and was compared with that of control. This experiment was performed on fluconazole sensitive isolates (standard, *n* = 3; clinical, *n* = 11). Experiments were performed thrice in replicate on separate days. Values were shown in terms of mean ± standard error of mean (SEM) of all three respective categories.

### 4.4. Confocal Scanning Laser Microscopy

Confocal scanning laser microscopy (CSLM) was used to evaluate the effect of test compounds on the architecture of yeast cells using propidium iodide (PI). PI is a fluorescent probe used to study the effect of drugs on membranes. It only penetrates cells with severe membrane lesions, showing increased red fluorescence. Cells (10^6^/mL) were incubated at 37 °C up to mid-exponential phase and were then treated with four times MIC of test compounds along with negative (no compound) and positive (2 mg/L Amphotericin B) controls. The samples were then inoculated at 37 °C for 30 min in dark. Unstained cells were always included as auto-fluorescence controls. The suspensions were centrifuged, washed and resuspended in PBS. Five microlitres of PI were added to the cell suspensions in order to obtain a final concentration of 1 μg/mL to determine the general and nuclear morphology, respectively. The cells were examined with Olympus Laser Confocal Scanning Microscope equipped with green helium neon laser (543 nm) for PI. The objective used was an oil immersion C-Apochromat lens (20×). Image acquisition was done by FV10-ASW1.6 Software.

### 4.5. Sterol Quantitation Assay

Total intracellular sterols were extracted as reported earlier with slight modifications [13]. Briefly, a single *Candida* colony from an overnight sabouraud dextrose agar plate culture was used to inoculate 50 mL of sabouraud dextrose broth (HiMedia) containing MIC and ½ MIC of all the test compounds along with negative control (without test compound) and positive control (fluconazole). The cultures were incubated for 16 h with shaking at 37 °C. The stationary-phase cells were harvested by centrifugation at 2700 rpm (Sigma 3K30) for 5 min and washed once with sterile distilled water. The net wet weight of the cell pellet was determined. Three milliliters of 25% alcoholic potassium hydroxide solution (25 g of KOH and 35 mL of sterile distilled water, brought to 100 mL with 100% ethanol), was added to each pellet and vortex mixed for 1 min. Cell suspensions were transferred to 16 mm × 100 mm sterile borosilicate glass screw-cap tubes and were incubated in an 85 °C water bath for 1 h. Following incubation, tubes were allowed to cool to room temperature. Sterols were then extracted by addition of a mixture of 1 mL of sterile distilled water and 3 mL of *n*-heptane followed by vigorous vortex mixing for 3 min. The heptane layer was transferred to a clean borosilicate glass screw-cap tube and stored at −20 °C for 24 h. Prior to analysis, an aliquot (1 mL) of sterol extract was diluted fivefold in 100% ethanol and scanned spectrophotometrically between 240 and 300 nm using LABOMED, INC Spectrophotometer (Culver City, CA, USA). The presence of ergosterol and the late sterol intermediate 24(28) DHE in the extracted sample resulted in a characteristic curve with four peaks (Figures 2 and 3). The absence of detectable ergosterol in extracts was indicated by a flat line. Ergosterol content was calculated as a percentage of the wet weight of the cell by the following Equations:

$$\begin{array}{c} \%\text{ergosterol }+\%24(28)\;\text{DHE}=[(A_{281.5}/290)\times F]/\text{pellet weight},\\ \%24(28)\;\text{DHE}=[(A_{230}/518)\times F]/\text{pellet weight},\\ \%\text{ergosterol}=[\%\text{ergosterol}+\%24(28)\;\text{DHE}]-\%24(28)\;\text{DHE} \end{array}$$

where *F* is the factor for dilution in ethanol and 290 and 518 are the *E* values (in percentages per centimetre) determined for crystalline ergosterol and 24(28) DHE, respectively. This experiment was performed on fluconazole sensitive isolates (standard, *n* = 3; clinical, *n* = 11) randomly selected out of total 14 isolates. Values were shown in terms of mean ± SEM of all three respective categories.

## 5. Conclusions

The present study achieved the excellent synthesis of a novel series of tetrazole ring bearing acyl hydrazone derivatives (**TH1**–**TH10**). This study confirmed the great potential of this new class of compounds as innovative antifungal agents. Through a preliminary SAR campaign, we found that presence and position of substituents decide the fungistatic and fungicidal nature of the compounds. Insight studies into mechanism suggest that the fungicidal activity resulted from extensive lesions of the plasma membrane. Target compounds also caused a considerable reduction in the amount of ergosterol. Unanswered concerns exist about the *in vivo* efficacy of the tested compounds. Future research towards this objective, based on animal models, may resolve these issues.

## Figures and Tables

**Table 1:** Minimum Inhibitory Concentrations of the compounds under study.

	Mean MIC/MFC (mg L^−1^)					
	
Compound	Standard lab strains (*n* = 3)		Clinical strains (*n* = 11)		Resistant strains (*n* = 3)	
			
	MIC	MFC	MIC	MFC	MIC	MFC
A1	64	na	64	na	-	-
A2	na	na	na	na	-	-
A3	na	na	na	na	-	-
A4	na	na	na	na	-	-
A5	na	na	na	na	-	-
TH1	6	na	6	na	12	64
TH2	na	na	na	na	na	na
TH3	62	128	62	128	128	256
TH4	4	16	8	16	4	16
TH5	8	64	16	64	16	128
TH6	4	64	8	128	4	128
TH7	16	32	16	32	64	64
TH8	2	na	8	na	64	na
TH9	4	na	4	na	32	na
TH10	32	na	32	na	64	na

Fluconazole MIC range: 4–64 mg L^−1^; Amphotericin B MIC range: 0.125–2 mg L^−1^; “na”: Inactive; “-”: Not tested.

**Table 2:** Isolates used in the present study.

Classification of isolates standard, *n* = 3	Type of isolate
ATCC 10261	*C. albicans*
ATCC 90028	*C. albicans*
ATCC 750	*C. tropicalis*
**Clinical susceptible**, *n* = 11	
Candidemia, VVC *, UTI (*n* = 6)	*C. albicans* (3), *C. tropicalis* (1), *C. glabrata* (1), *C. krusei* (1)
Cutaneous (*n* = 3)	*C. albicans* (2), *C. tropicalis* (1)
Oropharyngeal (*n* = 2)	*C. albicans* (1), *C. tropicalis* (1)
**Clinical Resistant**, *n* = 3	
Candidemia, VVC *	*C. albicans* (1), *C. glabrata* (1), *C. krusei* (1)

* VVC, Vulvovaginal candidiasis; UTI, Urinary tract infections, in HIV and non-HIV patients.

**Table 3:** Effect of the test compounds on ergosterol biosynthesis in different fluconazole-sensitive and fluconazole-resistant *Candida* isolates.

Test compounds −ive Control	Mean ergosterol content * of susceptible *Candida* cells 0.0181 ± 0.0045		Mean ergosterol content * of resistant *Candida* cells 0.0201 ± 0.0034
**TH1**	½ MIC	0.0135 ± 0.0013(25) **	0.0115 ± 0.0012 (43) **
	MIC	0.00012 ± 0.0001(99) **	0.0009 ± 0.0004 (96) **
**TH2**	½ MIC	0.0167 ± 0.0034(8) **	0.0171 ± 0.0021 (15) **
	MIC	0.0107 ± 0.0079 (41) **	0.0055 ± 0.001 (73) **
**TH3**	½ MIC	0.0087 ± 0.002(52) **	0.0093 ± 0.0024 (54) **
	MIC	0.00011 ± 0.0001(99) **	0.00149 ± 0.00012 (93) **
**TH4**	½ MIC	0.0027 ± 0.0028 (85) **	0.0061 ± 0.0034 (70) **
	MIC	0 ± 0 (100) **	0 ± 0 (100) **
**TH5**	½ MIC	0.0059 ± 0.0034(67) **	0.0049 ± 0.0011 (76) **
	MIC	0.00044 ± 0.0002(97) **	0.00039 ± 0.0001(98) **
**TH6**	½ MIC	0.0046 ± 0.0017(75) **	0.0122 ± 0.001 (40) **
	MIC	0.00041 ± 0.0002(98) **	0 ± 0 (100) **
**TH7**	½ MIC	0.0066 ± 0.003(63) **	0.0109 ± 0.001 (46) **
	MIC	0.0006 ± 0.0003(96) **	0.0022 ± 0.0011 (89) **
**TH8**	½ MIC	0.0062 ± 0.0019(66) **	0.014 ± 0.004 (30) **
	MIC	0.00018 ± 0.0001(99) **	0 ± 0 (100) **
**TH9**	½ MIC	0.0127 ± 0.0024(29) **	0.0102 ± 0.01 (50) **
	MIC	0.00041 ± 0.0003(99) **	0.0028 ± 0.0004 (86) **
**TH10**	½ MIC	0.0144 ± 0.0028(21) **	0.0089 ± 0.0008 (56) **
	MIC	0.00052 ± 0.0003(97) **	0.001 ± 0.0003(95) **
**A1**	124 mg L^−1^	0.0045 ± 0.0011 (75) **	-
**A2**	124 mg L^−1^	0.00016 ± 0.0001(49) **	-
**A3**	124 mg L^−1^	0.00010 ± 0.0001(99) **	-
**A4**	124 mg L^−1^	0.00039 ± 0.0001(98) **	-
**A5**	124 mg L^−1^	0.00044 ± 0.0002(97) **	-
**+ive Control**	0 ± 0 (100) **		0.0147 (27) **

* Expressed as a percentage of the wet weight of the cell ± standard error of the mean (followed in parentheses by the percent reduction in the mean cellular ergosterol content compared with that of control cells grown without test compounds);

** Significant reduction compared with controls (*p* < 0.05) after Student’s *t* test.
